# Effects of Sodium Glucose Cotransporter 2 (SGLT2) Inhibitors on Lipid Profiles in Type 2 Diabetes: A Systematic Review

**DOI:** 10.7759/cureus.102618

**Published:** 2026-01-30

**Authors:** Mushrega Abdalla, Mudathir Elyas Suleiman Khamees, Ahmed-Lamin Gehani, Abubaker Ibrahim Mohammed Ibrahim, Muntassir Y Yousif, Noor Kheir, Safa Mohammed Qasem Alqasem, Asim Ahmed

**Affiliations:** 1 Medicine and Surgery, University of Khartoum, Khartoum, SDN; 2 Internal Medicine, Faculty of Medicine and Surgery, Alzaiem Alazhari University, Port Sudan, SDN; 3 Family Medicine, Near East University, Nicosia, CYP; 4 Medicine, Al Dhafra Hospitals, Al Dhafra, ARE; 5 Internal Medicine, University of Gezira, Wad Madani, SDN; 6 Neuroscience, Faculty of Science, University of Alberta, Alberta, CAN; 7 General Practice, Al Safaa Pioneer Clinic, Jeddah, SAU; 8 Epidemiology and Public Health, University of Gezira, Wad Madani, SDN

**Keywords:** diabetes mellitus type 2, dyslipidemia, lipid metabolism, sodium-glucose cotransporter 2 inhibitors, systematic review

## Abstract

Sodium glucose cotransporter 2 (SGLT2) inhibitors provide cardiometabolic benefits in adults with type 2 diabetes mellitus, yet their effects on lipid metabolism are reported inconsistently across clinical trials and mechanistic studies and may vary with background statin therapy and differences in lipid measurement and reporting. We conducted a systematic review to evaluate the impact of SGLT2 inhibitors on conventional lipid parameters first and selected advanced lipid markers second in adults with type 2 diabetes. We searched multiple databases using predefined eligibility criteria and extracted data in duplicate. We assessed the risk of bias using design-appropriate tools and synthesized findings qualitatively because heterogeneity limited quantitative pooling. Overall, evidence across included studies suggests that SGLT2 inhibitors are essentially neutral with respect to low-density lipoprotein cholesterol (LDL-C), with generally favorable directions for high-density lipoprotein cholesterol (HDL-C) and triglycerides in some study settings. At the same time, findings on qualitative lipoprotein remodeling and advanced lipid phenotypes remain heterogeneous. These results support a lipid neutral to mildly favorable profile on standard lipid panels and emphasize the need for standardized reporting of advanced lipid measures and longer follow-up to clarify clinical relevance.

## Introduction and background

Type 2 diabetes mellitus is a global epidemic affecting hundreds of millions of adults worldwide and is projected to rise substantially in the coming decades [1]. Beyond chronic hyperglycemia, this condition is associated with a high burden of cardiovascular and renal complications, highlighting the importance of comprehensive risk factor management [2]. A characteristic pattern of diabetic dyslipidemia includes elevated triglyceride levels, reduced high-density lipoprotein cholesterol (HDL-C) levels, and a predominance of small dense low-density lipoprotein (LDL) particles, which accelerates atherosclerosis and contributes to residual cardiovascular risk [3].

Sodium glucose cotransporter 2 (SGLT2) inhibitors, including empagliflozin, dapagliflozin, and canagliflozin, are oral therapies for type 2 diabetes mellitus that lower blood glucose by inhibiting renal tubular glucose reabsorption [4], and large cardiovascular outcome trials, including the CANVAS Program and DECLARE TIMI 58, demonstrated reductions in hospitalization for heart failure and favorable renal outcomes; CANVAS also reduced major adverse cardiovascular events, whereas DECLARE TIMI 58 met the noninferiority for major adverse cardiovascular events [5,6]. Collectively, these data contributed to positioning SGLT2 inhibitors as cardiometabolic therapies with benefits extending beyond glycemic control [2,4].

Given that many candidates for SGLT2 inhibitors also meet indications for statin therapy and other lipid-modifying strategies, and that background statin and other lipid-modifying therapies may mask or modify observed lipid changes, clarifying the drugs' effect on atherogenic lipid profiles is of direct clinical importance to support interpretation of trial lipid results [3,7]. Systematic reviews and meta-analyses of randomized trials have evaluated overall benefits and harms of SGLT2 inhibitors and reported associated changes in cardiometabolic parameters [8]. More lipid focused meta analyses have reported modest shifts in standard lipid measures, including low-density lipoprotein cholesterol (LDL-C) and HDL-C, following SGLT2 inhibitor initiation, but they variably capture advanced lipid markers, including apolipoprotein B (apoB), remnant cholesterol, lipoprotein subfractions, HDL function, and lipidomic or qualitative remodeling signals, raising questions about interpretation in already high-risk populations [9]. Mechanistic reviews propose several pathways that could underlie these lipid changes, including altered substrate utilization and downstream effects on hepatic lipoprotein production and clearance [10].

Given the central role of dyslipidemia in driving cardiovascular risk in type 2 diabetes mellitus and the mixed directionality of reported lipid changes, and the variable reporting of advanced lipid markers across studies, a focused synthesis is warranted [3,7]. This systematic review aimed to summarize the effects of SGLT2 inhibitors on LDL-C, HDL-C, triglycerides, and total cholesterol and to describe reported advanced lipid markers when available, in adults with type 2 diabetes mellitus.

Objectives

We conducted a systematic review to evaluate the effects of SGLT2 inhibitors on standard serum lipid outcomes, including LDL-C, HDL-C, triglycerides, and total cholesterol, in adults with type 2 diabetes mellitus. Secondary objectives were to compare lipid effects across individual SGLT2 agents and to summarize advanced lipid markers and mechanistic explanations proposed in the literature [10,11]. We hypothesized that SGLT2 inhibitors have neutral to modest effects on standard lipid parameters and that these changes would not negate established cardiovascular and renal benefits reported in major outcome trials [5,6,8], with additional supportive evidence from experimental studies on diabetes associated renal and hepatic fibrosis [11].

## Review

Methods

This systematic review, reported in accordance with the Preferred Reporting Items for Systematic Reviews and Meta-Analyses (PRISMA) 2020 statement [12], evaluated the effects of sodium glucose cotransporter 2 inhibitors on lipid metabolism in adults with type 2 diabetes mellitus using the PICOS framework.

Protocol and Registration

The protocol was developed using the PICOS framework and prospectively registered in PROSPERO (ID 1237510) on 21 November 2025, titled "The Effects of SGLT2 Inhibitors on Lipid Metabolism: A Systematic Review and Meta-Analysis." The protocol specified eligibility criteria, the search strategy, outcomes, and the planned approach to evidence synthesis. No formal sample size or power calculation was performed because the number of participants was determined by the available eligible studies.

Eligibility Criteria

The population of interest was adults aged 18 years or older with type 2 diabetes mellitus, with or without comorbid cardiovascular disease, heart failure, nephropathy, obesity, metabolic syndrome, or nonalcoholic fatty liver disease, provided that lipid outcomes were reported. Interventions included any sodium glucose cotransporter 2 inhibitor, for example, empagliflozin, dapagliflozin, canagliflozin, or ertugliflozin, at any approved dose. Comparators were placebo, usual care, or active non-sodium glucose cotransporter 2 glucose-lowering therapies, such as dipeptidyl peptidase 4 inhibitors, glucagon-like peptide 1 receptor agonists, or insulin-based regimens.

The primary outcomes were changes in conventional lipid parameters, including LDL-C, HDL-C, triglycerides, and total cholesterol, reported as absolute change, percentage change from baseline, or between-group difference. Secondary outcomes included changes in body weight and anthropometric indices, glycaemic markers including fasting plasma glucose and HbA1c, indices of insulin resistance, and mechanistic or lipidomic measures, for example, low-density lipoprotein subfractions, high-density lipoprotein subtypes, and angiopoietin-like protein 3, where available.

Eligible designs were randomized controlled trials and prospective or retrospective observational cohorts that provided extractable quantitative lipid data before and after sodium glucose cotransporter 2 inhibitor therapy. Case reports, case series, narrative reviews, editorials, nonhuman studies, and reports without extractable lipid outcomes were excluded. Only full-text articles in English were included, with no date restrictions. Eligibility criteria are summarized using the PICOS framework (see Table 1).

**Table 1:** PICOS framework and eligibility criteria PICOS = Population, Intervention, Comparator, Outcomes, Study design; LDL-C = low-density lipoprotein cholesterol; HDL-C = high-density lipoprotein cholesterol; NAFLD/MASLD = non-alcoholic fatty liver disease/metabolic dysfunction-associated steatotic liver disease; SGLT2 = sodium-glucose cotransporter-2 (derived from the manuscript in the Methods section).

PICOS element	Definition in this review
Population (P)	Adults (≥18 years) with type 2 diabetes mellitus, with or without comorbid cardiovascular disease, heart failure, nephropathy, obesity, metabolic syndrome, or NAFLD/MASLD, provided lipid outcomes were reported.
Intervention (I)	Any SGLT2 inhibitor at any approved dose (e.g., empagliflozin, dapagliflozin, canagliflozin, ertugliflozin; others where eligible).
Comparator (C)	Placebo, usual care, or active non-SGLT2 glucose-lowering therapy (e.g., DPP-4 inhibitors, GLP-1 receptor agonists, insulin-based regimens).
Outcomes (O) – Primary	Changes in LDL-C, HDL-C, triglycerides, and total cholesterol (absolute change, % change from baseline, or between-group difference).
Outcomes (O) – Secondary	Body weight/anthropometrics; glycaemic markers (fasting plasma glucose, HbA1c); insulin resistance indices; advanced/mechanistic lipid outcomes where available (e.g., LDL subfractions, HDL subtypes, ANGPTL3, ApoB48).
Study design (S)	Randomized controlled trials and prospective/retrospective observational cohort studies with extractable quantitative lipid data pre/post SGLT2 inhibitor therapy.
Exclusions	Case reports/series, narrative reviews/editorials, nonhuman studies, and reports without extractable lipid outcomes; non-English; non-full text.
Timeframe	No date restrictions (database inception to August 2025).

Information Sources and Search Strategy

We searched MEDLINE via PubMed, Embase, Web of Science, and the Cochrane Library from database inception to August 2025. The search combined controlled vocabulary and free text terms relating to SGLT2 inhibition, type 2 diabetes mellitus, and lipid outcomes (e.g., empagliflozin, cholesterol, triglycerides). Titles, abstracts, and keywords were searched, and reference lists of key randomized trials and relevant systematic reviews were screened manually to identify additional studies. No language limits were applied during the search, but eligibility was restricted to studies with full text available in English.

Conference abstracts from major diabetes and cardiology meetings from 2023 to 2025 were also screened. Where possible, corresponding full-text publications were retrieved. Abstracts without sufficient quantitative data were used only for contextualisation and were not included in the primary synthesis. All records were imported into reference management software, and duplicates were removed before screening. The full search strategy is presented in Table 2.

**Table 2:** Information sources and search strategy The search combined controlled vocabulary and free-text terms related to SGLT2 inhibition, T2DM, and lipid outcomes, with supplementary manual screening and conference abstract checks, as described in the Methods section.

Component	Details
Databases searched	MEDLINE (via PubMed), Embase, Web of Science, Cochrane Library
Coverage	From database inception to August 2025
Core concepts combined	(1) SGLT2 inhibitors, (2) type 2 diabetes mellitus, (3) lipid outcomes
Keywords (examples reported)	“sodium glucose cotransporter 2”, “SGLT2 inhibitor”, empagliflozin, dapagliflozin, canagliflozin, ertugliflozin; “type 2 diabetes”; cholesterol, LDL, HDL, triglycerides, dyslipidaemia
Search fields	Titles/abstracts/keywords; controlled vocabulary where applicable
Other sources	Manual screening of reference lists of key RCTs and relevant systematic reviews; conference abstracts from major diabetes/cardiology meetings (2023–2025), used for context if quantitative data were insufficient
Record handling	Imported into reference management software; duplicates removed before screening

Study Selection

Study selection occurred in two stages. First, two reviewers independently screened titles and abstracts and excluded clearly irrelevant reports, including studies of nondiabetic populations, non-sodium glucose cotransporter 2 interventions, articles without lipid outcomes, and non-original research. Second, full-text articles of potentially eligible records were assessed against the predefined inclusion and exclusion criteria. Disagreements were resolved by discussion, with a third reviewer consulted when needed.

Data Extraction and Management

Two reviewers independently extracted data using a piloted, standardised form. For each study, we recorded author and year, country and clinical setting, study design, sample size, and baseline characteristics, including age, sex, duration of type 2 diabetes mellitus, and presence of cardiovascular disease, heart failure, nephropathy, or nonalcoholic fatty liver disease. We also extracted details of sodium glucose cotransporter 2 inhibitor type and dose, comparator regimen including placebo, usual care, or active non-sodium glucose cotransporter 2 therapy, and duration of follow-up.

For lipid outcomes, we extracted baseline and follow-up values for total cholesterol, LDL-C, HDL-C, and triglycerides, along with reported mean absolute changes, percentage changes, or between-group differences, and corresponding measures of variability, including standard deviation, standard error, or confidence interval, when available. All values were converted to milligrams per decilitre using standard factors. For total cholesterol, LDL-C, and HDL-C, mg per dL equals mmol per L multiplied by 38.67. For triglycerides, mg per dL equals mmol per L multiplied by 88.57.

Where reported, we also collected data on lipid subfractions, postprandial lipid responses, and mechanistic biomarkers such as angiopoietin-like protein 3 and ApoB48. When outcomes were available only as figures, we estimated numerical values from graphs where feasible. If key data were unclear or missing, we checked the main text and supplementary files and, where necessary, attempted to contact study authors.

Studies with insufficient data for a particular lipid parameter were included in the qualitative synthesis but omitted from outcome-specific analyses for that parameter. No statistical imputation of missing lipid values or measures of variability was performed.

Risk of Bias Assessment

Risk of bias was assessed using design-appropriate tools. Randomized and interventional studies were evaluated using the Cochrane Risk of Bias 2 (RoB 2) tool (including the RoB 2 variant for crossover trials when applicable) [13]. Judgments were made across five domains: D1 randomization process, D2 deviations from intended interventions, D3 missing outcome data, D4 measurement of the outcome, and D5 selection of the reported result. Each domain was rated as low risk, some concerns, or high risk, and an overall RoB 2 judgment was derived from the domain-level ratings.

Nonrandomized comparative studies of interventions were assessed using the ROBINS I tool [14], which evaluates bias due to confounding, participant selection, intervention classification, deviations from intended interventions, missing data, outcome measurement, and selective reporting. Overall, ROBINS I judgments were categorized as low, moderate, serious, or critical risk of bias.

For uncontrolled single-arm pre-post studies without a concurrent comparator, we used an adapted domain-based assessment informed by ROBINS I signaling domains [14], with particular emphasis on confounding and time-varying cointerventions, and findings were interpreted cautiously because ROBINS I is primarily intended for nonrandomized comparative studies. Systematic reviews and meta-analyses were appraised separately using AMSTAR 2, and protocol and rationale papers were listed for transparency but were not eligible for outcome-level risk of bias assessment. Assessments were conducted independently by two reviewers, with disagreements resolved by consensus or by consultation with a third reviewer.

Data Synthesis

We synthesized findings qualitatively due to variability in study designs, comparators, follow-up durations, and outcome reporting across included studies. Where studies reported comparable outcomes, we summarized the direction and consistency of changes in total cholesterol, LDL-C, HDL-C, and triglycerides. We also summarized mechanistic and lipidomic outcomes narratively when reported.

Results

Study Selection and Characteristics

**Figure 1:**
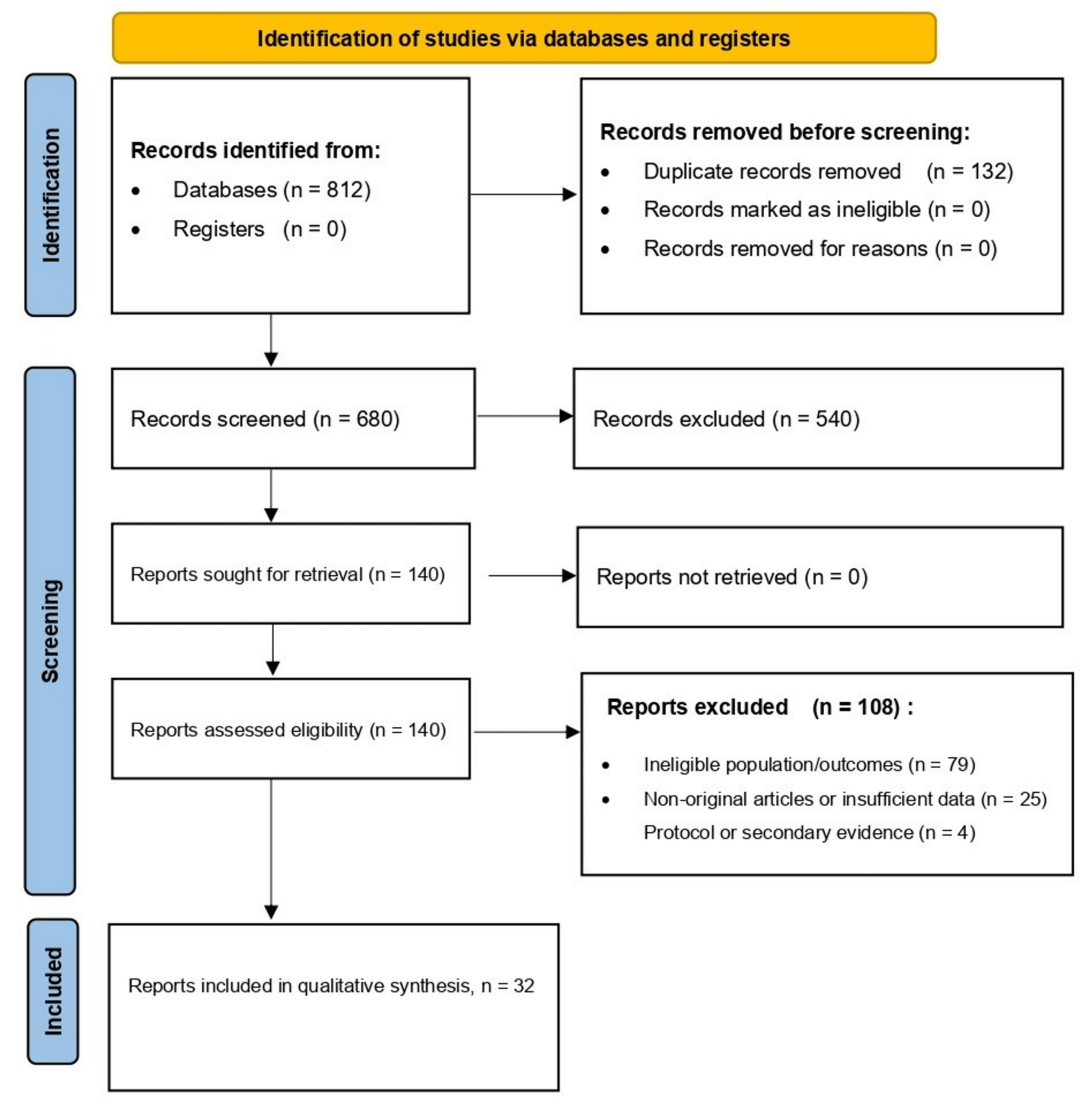
PRISMA flow diagram of study identification, screening, eligibility assessment, and inclusion Records were identified through database searching; duplicates were removed before screening. Titles and abstracts were screened, and full texts of potentially eligible reports were assessed against predefined inclusion and exclusion criteria. Studies meeting eligibility criteria were included in the qualitative synthesis. PRISMA: Preferred Reporting Items for Systematic Reviews and Meta-Analyses

The systematic database search identified 812 records. After removal of duplicates (n = 132), 680 records were screened by title and abstract, and 540 were excluded. Full-text review was conducted for 140 reports, of which 104 were excluded due to ineligible population, intervention, or outcomes (n = 79), or because reports were non-original or had insufficient data (n = 25). Ultimately, 32 primary studies met the inclusion criteria and were included in the qualitative synthesis. Four additional records were evidence syntheses or protocol and design papers that were retained for context and transparency, but were not treated as included studies for outcome level synthesis (see Figure 1).

Records were identified through database searching; duplicates were removed before screening. Titles and abstracts were screened, and full texts of potentially eligible reports were assessed against predefined inclusion and exclusion criteria. Studies meeting eligibility criteria were included in the qualitative synthesis.

Of the 32 included primary studies, 23 were interventional studies [15-37], and nine were observational real-world nonrandomized studies [38-46]. Secondary evidence and protocol or design records were listed separately for transparency and context and were not included in outcome synthesis [8,47-49]. Collectively, these investigations enrolled more than 30,000 adults with type 2 diabetes mellitus. Follow-up ranged from short mechanistic investigations (~4 weeks) [16] to longer follow-up of approximately three years [19,40].

The most frequently evaluated SGLT2 inhibitors were empagliflozin, dapagliflozin, and canagliflozin, whereas ipragliflozin and luseogliflozin were examined less often. Comparators included placebo, standard of care, and active glucose-lowering therapies, most commonly DPP-4 inhibitors (see Table 3).

**Table 3:** Interventional studies (randomized, crossover, or interventional comparative; N = 23) RCT, randomized controlled trial; T2DM, type 2 diabetes mellitus; LDL-C, low-density lipoprotein cholesterol; HDL-C, high-density lipoprotein cholesterol; TG, triglycerides; TC, total cholesterol; NR, not reported

Authors (Year)	Study Design	Population	SGLT2 Intervention	Comparator	N	Duration	Key Lipid Changes (LDL-C, HDL-C, TG, TC)	Mechanistic or Key Findings to Cite	Country
Kahl et al. (2020) [15]	Randomized, double-blind, placebo-controlled, phase 4	Well-controlled type 2 diabetes mellitus	Empagliflozin	Placebo	Not reported	Not reported	Not primarily a lipid trial	Empagliflozin lowers liver fat content	Germany
Lauritsen et al. (2022) [16]	Randomized, double blind, crossover	Type 2 diabetes mellitus on metformin	Empagliflozin 10 mg daily	Placebo	13	4 weeks	Not extracted	Increased visceral adipose tissue free fatty acid uptake and reduced GLUT4 expression in abdominal subcutaneous adipose tissue	Denmark
Fadini et al. (2017) [17]	Randomized, placebo controlled	Type 2 diabetes mellitus	Dapagliflozin 10 mg	Placebo	33 randomized, 31 completed	12 weeks	Not extracted	Focus on HDL function and cholesterol efflux capacity, no consistent changes after adjustment	Italy
Hayashi et al. (2017) [18]	Randomized, prospective, single center	Type 2 diabetes mellitus on oral agents	Dapagliflozin	Sitagliptin	80, 40 per group	12 weeks	Reduced small dense LDL-C, increased HDL2 C, reduced TG, values not extracted	Shift in LDL particle profile and HDL subfractions with dapagliflozin compared with sitagliptin	Japan
Zinman et al. (2015) [19]	Randomized controlled trial, cardiovascular outcome trial, EMPA REG OUTCOME	Type 2 diabetes mellitus at high cardiovascular risk	Empagliflozin 10 mg or 25 mg daily	Placebo	7020	Median 3.1 years	Small increases in LDL-C and HDL-C, values not extracted	Reduced cardiovascular events and mortality	Multinational
Bauer et al. (2025) [20]	Randomized, double blind, placebo controlled, parallel group, phase 4	Type 2 diabetes mellitus with heart failure phenotype, EmDia	Empagliflozin 10 mg daily	Placebo	144	1 week and 12 weeks time points	Lipidomics primarily standardizes lipids, not primary	Distinct lipidome signatures after empagliflozin at 1 week and 12 weeks	Germany
Emanuelsson et al. (2025) [21]	Post hoc analyses of two double blind, placebo controlled trials, Empire HF and SIMPLE	Heart failure with reduced ejection fraction cohort and type 2 diabetes mellitus cohort	Empagliflozin 10 mg or 25 mg	Placebo	190 and 90	12 weeks	No material changes in lipids and lipoproteins versus placebo	Supports cardioprotection independent of lipid concentration changes	Multicenter
Bays et al. (2017) [22]	Post-hoc pooled analysis of phase 3 placebo-controlled trials	Type 2 diabetes mellitus with and without high TG and low HDL phenotype	Dapagliflozin 10 mg	Placebo	Not reported	24 weeks	Minor increases in LDL-C and HDL-C, TG variable, values not extracted	Lipid effects broadly similar across baseline lipid phenotype groups	Multinational
Dixon et al. (2021) [23]	Post-hoc analysis of CANA HF randomized trial	Type 2 diabetes mellitus with heart failure with reduced ejection fraction	Canagliflozin 100 mg daily	Sitagliptin 100 mg daily	36 enrolled, 35 analyzed	12 weeks	No significant between-group differences in LDL-C, HDL-C, TG, TC	Canagliflozin did not increase LDL-C compared with sitagliptin in this cohort	United States
Liu et al. (2024) [24]	Prospective interventional study	Type 2 diabetes mellitus	Canagliflozin, dose not reported	Metformin	Not reported	12 weeks	HDL-C increased, LDL-C and TG trend lower, values not extracted	ANGPTL3 reduction proposed as a mechanism linked to HDL changes	China
Hiruma et al. (2021) [25]	Randomized, open label, blinded endpoint, parallel group, ASSET	Early stage type 2 diabetes mellitus	Empagliflozin	Sitagliptin	44	12 weeks	HDL-C increased, full lipid table not extracted	Ketone bodies increased and insulin resistance markers improved	Japan
Burggraaf et al. (2022) [26]	Randomized, double blind, placebo controlled, proof of concept	Male type 2 diabetes mellitus on intensive insulin	Dapagliflozin 10 mg	Placebo	14	12 weeks	No effect on fasting or postprandial cholesterol or triglycerides	Reduced chylomicron remnants and ApoB48, increased postprandial ketones and glucagon	Netherlands
Wazir et al. (2022) [27]	Interventional clinical study	Type 2 diabetes mellitus	Empagliflozin 10 mg versus 25 mg	Dose comparison	59	12 weeks	Not extracted	Lipid profile outcomes reported, details not extracted	Pakistan
Jojima et al. (2021) [28]	Interventional clinical study	Adults with type 2 diabetes mellitus	Empagliflozin 10 mg daily	Control not reported	51	12 weeks	Not extracted	Increased campesterol, a marker of cholesterol absorption	Japan
Najim et al. (2025) [29]	Comparative clinical study	Type 2 diabetes mellitus with diabetic nephropathy	Dapagliflozin versus empagliflozin	Active comparator	41	16 weeks	Not extracted	Nephropathy-focused, lipid outcomes used only if extractable	Palestine
Fukada et al. (2025) [30]	Clinical study in metabolic dysfunction associated with steatotic liver disease with type 2 diabetes mellitus	Metabolic dysfunction associated with steatotic liver disease with type 2 diabetes mellitus	Dapagliflozin	Comparator not reported	Not reported	Not reported	Not extracted	Effectiveness and risks in metabolic dysfunction associated with steatotic liver disease population	Japan
Langslet et al. (2020) [31]	Post hoc analysis of EMPA REG OUTCOME dataset	Type 2 diabetes mellitus with cardiovascular risk	Empagliflozin 10 mg or 25 mg daily	Placebo	7020	About 3.1 years	Not a lipid change analysis	Outcomes examined in relation to achieved LDL-C strata	Multinational
Moura et al. (2021) [32]	Post hoc subgroup analysis of DECLARE TIMI 58, conference abstract	Type 2 diabetes mellitus with metabolic syndrome	Dapagliflozin 10 mg daily	Placebo	17160	Variable follow-up	Not extracted	Metabolic syndrome subgroup analysis	Multinational
Li et al. (2025) [33]	Interventional study	Overweight or obese type 2 diabetes mellitus	Dapagliflozin plus probiotics	Dapagliflozin alone	120	24 weeks	Not extracted	Gut microbiota and metabolic syndrome context	China
Kabir et al. (2023) [34]	Randomized, placebo-controlled trial	Adults with type 2 diabetes mellitus and dyslipidaemia	Dapagliflozin 10 mg daily added to existing therapy	Placebo added to existing therapy	26	12 weeks	LDL-C decreased, HDL-C increased, TG decreased, TC not reported	Primary outcome was the change in lipid profile after 12 weeks; secondary outcomes included improved HbA1c, fasting plasma glucose, weight, and systolic blood pressure	Pakistan
Nagao et al. (2024) [35]	Randomized trial, SUCRE	Early-phase type 2 diabetes mellitus	Ipragliflozin 50 mg daily	Sitagliptin 50 mg daily	160	6 months	Not extracted	Atherogenic lipoprotein profile changes	Japan
Rau et al. (2021) [36]	Randomized, placebo-controlled	Type 2 diabetes mellitus	Empagliflozin	Placebo	Not reported	12 weeks	Not extracted	Lipoprotein subfraction changes	Germany
Ejiri et al. (2022) [37]	Randomized, open-label, controlled	Type 2 diabetes mellitus with heart failure	Luseogliflozin 2.5 mg daily	Voglibose	173	12 weeks	Not extracted	High-risk lipid profiles and inflammatory markers	Japan

Key Lipid Profile Findings

Triglycerides and HDL-C, modest improvements with variable consistency: Across interventional studies, SGLT2 inhibitors showed small, heterogeneous effects on standard fasting lipids. In pooled dapagliflozin analyses, lipid changes were small and appeared to vary by baseline phenotype, and a post-hoc comparison found no significant between-group differences in conventional serum lipids for canagliflozin versus sitagliptin. Mechanistic and lipid-focused trials described qualitative lipoprotein shifts, including reductions in small dense LDL-C and changes in HDL subfractions, and lipidomic profiling suggested broader remodeling not fully captured by routine fasting lipid panels. Evidence syntheses likewise describe modest HDL-C increases and triglyceride reductions, with variability across trials [18,20,22,23,47].

These patterns were broadly echoed in nonrandomized settings, but interpretation is limited by confounding and changes in background therapy over follow-up (see Table 4).

**Table 4:** Observational, real-world, and nonrandomized studies (N = 9) T2DM, type 2 diabetes mellitus; LDL-C, low-density lipoprotein cholesterol; HDL-C, high-density lipoprotein cholesterol; TG, triglycerides; TC, total cholesterol; NR, not reported; NS, not statistically significant

Authors (Year)	Study Design	Population	SGLT2 Intervention	Comparator	N	Duration	Key Lipid Changes (LDL-C, HDL-C, TG, TC)	Mechanistic Findings	Country
Kamijo et al. (2019) [38]	Multicenter prospective study	Adults with type 2 diabetes mellitus	Canagliflozin 100 mg daily	None, single arm	22	12 weeks	LDL-C not reported, HDL-C increased in very large and large HDL fractions, TG not reported, TC not reported	Increased cholesterol in specific HDL fractions, weight and blood pressure reduced	Japan
Doğanay et al. (2023) [39]	Retrospective comparative study	Newly diagnosed hypertensive type 2 diabetes mellitus	Sodium glucose cotransporter 2 inhibitor users, agents compared, including canagliflozin subgroup	Non sodium glucose cotransporter 2 inhibitor controls	236	Not reported	LDL-C not reported, HDL-C not reported, TG not reported, TC not reported	Pre- and post-lipid changes assessed, subgroup analyses reported	Turkey
Ku et al. (2021) [40]	Prospective observational study	Adults with type 2 diabetes mellitus on quadruple therapy	Empagliflozin versus dapagliflozin	Head to head groups	362	3 years	Not extracted	Long term effectiveness and safety, lipid benefit noted	South Korea
Grubić Rotkvić et al. (2023) [41]	Prospective nonrandomized observational study	Type 2 diabetes mellitus, myocardial dysfunction focus	Sodium glucose cotransporter 2 inhibitor, unspecified	Comparator group, nonrandomized	64	6 months	Lipid profile changed significantly, direction not stated	Metabolic parameters and diastolic function markers improved	Croatia
Khanna et al.(2025) [42]	Prospective open label parallel group study	Uncontrolled type 2 diabetes mellitus on metformin	Dapagliflozin	Vildagliptin	383 enrolled, 248 completed	24 weeks	LDL-C increased, HDL-C increased, TG no significant between group difference, TC decreased	Between group pattern reported for LDL-C, HDL-C, and TC, values not extracted	India
Lim et al.(2022) [43]	Retrospective propensity score matched cohort	Type 2 diabetes mellitus without prior cardiovascular disease or chronic kidney disease	Dapagliflozin versus empagliflozin	Matched control cohort included	3684	Median 43.4 months	LDL-C increased, particularly with empagliflozin, HDL-C not reported, TG not reported, TC not reported	Comparative real world outcomes, lipid signal mainly LDL-C	South Korea
Pahlavan et al. (2025) [44]	Prospective single center clinical trial	Type 2 diabetes mellitus with nonalcoholic fatty liver disease	Empagliflozin add on to metformin	None, single arm	80 enrolled, 74 completed	12 weeks	LDL-C decreased, HDL-C no significant change, TG decreased, TC decreased	Improved metabolic profile and liver enzymes, lipid improvements reported	Iran
Cokro et al. (2025) [45]	Real world comparative cohort, paired data analysis	Adults with type 2 diabetes mellitus	Dapagliflozin 10 mg daily	Empagliflozin 10 mg daily	Up to 319, variable by outcome	12 months	LDL-C decreased in both groups. HDL-C increased with dapagliflozin and no significant change with empagliflozin. TG decreased with dapagliflozin and no significant change with empagliflozin. TC decreased with empagliflozin and no significant change with dapagliflozin	Adjusted analyses showed broadly similar lipid patterns	Indonesia
Gogoleva et al. (2023) [46]	Prospective controlled study	Type 2 diabetes mellitus, metabolic focus	Dapagliflozin 10 mg daily	Standard management control	60	6 months	LDL-C decreased, HDL-C not reported, TG decreased, TC decreased	Improvements in fat and carbohydrate metabolism reported	Russia

LDL-C, heterogeneous patterns with mixed qualitative signals: The impact of sodium glucose cotransporter 2 inhibitors on LDL-C was heterogeneous across study designs and clinical contexts. In randomized and interventional studies, qualitative lipoprotein effects were not uniform. Some mechanistic evidence suggests reductions in small dense LDL-C and potentially favorable shifts in HDL subfractions [18]. In longer term outcome trials and related analyses, LDL-C generally remained unchanged or increased slightly, as reported in EMPA REG OUTCOME and associated analyses, and in DECLARE-related reporting within included sources [19,31,32]. Other studies reported no clear mean change in standard LDL measures [21] or increases in LDL-related markers without clear particle size improvement [36].

Across broader pooled evidence, meta-analyses have reported small increases in LDL-C alongside changes in HDL-C, with heterogeneity by dose, ethnicity, and drug type [8,9]. In real-world and pragmatic settings, LDL-C trajectories were variable and may reflect differences in baseline risk, weight change, glycaemic control, and background lipid therapy [43,45]. Comparative data also suggest that LDL-C direction can depend on the active comparator and therapeutic context; for instance, LDL-C increased with dapagliflozin relative to vildagliptin in an open-label comparative study [42].

Secondary findings and mechanistic insights: Several studies reported mechanistic signals that may underlie lipid modulation, and these findings are summarized here separately from the clinical lipid outcome synthesis. Changes in tissue-level lipid handling and transport were described in adipose-focused work [16]. Biomarker signals potentially relevant to lipid metabolism were reported, including angiopoietin-like protein 3 findings alongside HDL-related changes [24]. Altered cholesterol handling was suggested by changes in absorption markers [28]. In metabolic dysfunction-associated steatotic liver disease populations, dapagliflozin was associated with improvements in related parameters that may be relevant to lipid handling [30]. Clinical lipid outcomes are interpreted in the corresponding Results subsections, while this paragraph is limited to mechanistic observations. Secondary evidence, protocol, and design records are listed in Table 5.

**Table 5:** Characteristics of secondary evidence, rationale and design studies, and post-hoc analyses (N = 4) Characteristics of secondary evidence, rationale and design studies, and post-hoc analyses retained for context and transparency and not included in outcome level synthesis (N = 4). T2DM, type 2 diabetes mellitus; LDL-C, low-density lipoprotein cholesterol; HDL-C, high-density lipoprotein cholesterol; TG, triglycerides; TC, total cholesterol

Authors (Year)	Study Design	Population	SGLT2 Intervention	Comparator	N	Duration	Key Lipid Trends (LDL-C, HDL-C, TG, TC)	Key Notes	Country
Storgaard et al. (2016) [8]	Systematic review (evidence synthesis)	Type 2 diabetes mellitus trials	Sodium glucose cotransporter 2 inhibitors	Various comparators	Not reported	Varied	Varied	Comprehensive synthesis of benefits and harms across multiple trials	Multinational
Chen et al. (2021) [47]	Systematic review of randomized trials (evidence synthesis)	Adults with type 2 diabetes mellitus	Sodium glucose cotransporter 2 inhibitors (multiple agents)	Placebo or active control across trials	36 randomized trials	24 weeks	LDL-C slight increase, HDL-C increase, TG decrease, TC no significant change	Summary of lipid shifts and metabolic effects reported across trials	Multinational
Shigiyama et al. (2019) [48]	Trial protocol (rationale and design): ASSET	Adults with type 2 diabetes mellitus	Empagliflozin 10 mg daily	Sitagliptin 100 mg daily	Planned 44	Planned 12 weeks	Not applicable	Protocol focused on cardiac fat, cardiac function, and metabolic measures	Japan
Shigiyama et al. (2018) [49]	Trial protocol (rationale and design): DIVERSITY-CVR	Adults with type 2 diabetes mellitus	Dapagliflozin 10 mg daily	Sitagliptin 100 mg daily	340	24 weeks	Not applicable	Protocol designed to evaluate cardiovascular risk prevention endpoints	Japan

Synthesis of Lipid Findings

Favorable and more consistent changes - triglycerides and HDL-C: Across study designs, the most consistent signals were modest triglyceride reductions and modest HDL-C increases, although effects were heterogeneous. Evidence syntheses of randomized trials generally describe small, directionally favorable triglyceride changes with variability by agent, dose, and population, and similarly report modest HDL-C increases. Large outcome trials and pooled clinical trial analyses typically showed minor absolute lipid changes, but the triglyceride direction was commonly favorable [19,22]. In mechanistic and lipoprotein subfraction studies, HDL-related changes included HDL2 increases and increases in large HDL fractions [18,38].

Mechanistic evidence supports a metabolic shift with SGLT2 inhibition that may influence lipid handling through increased fatty acid utilization and broader metabolic adaptations, and lipidomic studies suggest remodeling of circulating lipid species not fully captured by standard fasting lipid measurements [4,10,20]. In observational cohorts, lipid changes should be interpreted cautiously because trajectories may be influenced by concurrent weight change, glycaemic improvement, and background lipid therapy over follow-up [45].

LDL-C findings: discrepancies by study design and inconsistent qualitative effects: Effects on LDL-C were heterogeneous and context dependent. Evidence syntheses of randomized trials generally describe small mean LDL-C increases or neutrality, with variability across agents and populations. Large long-term outcome trials commonly showed no significant LDL-C change or slight increases, consistent with broader randomized trial evidence [5,6,19].

Evidence for a consistently improved LDL phenotype was mixed. Some mechanistic work demonstrated reductions in small dense LDL-C [18]. However, detailed subfraction studies have reported discordant findings, including LDL-related increases without clear particle size improvement, or no meaningful lipid or lipoprotein concentration differences versus placebo [21,36].

Implications and mechanistic insights: Because diabetic dyslipidaemia reflects not only LDL-C concentration but also the triglyceride and HDL axis and lipoprotein quality, the overall pattern of modest triglyceride reductions, modest HDL-C increases, and variable LDL-C suggests that SGLT2 inhibitors may improve selected components of the atherogenic dyslipidaemia profile even when LDL-C is unchanged or slightly increased [3,8,9]. Qualitative lipid remodeling is inconsistent: favorable changes, including reductions in small dense LDL-C and increases in HDL2, and measures of HDL function in randomized designs, have been reported in mechanistic settings, whereas other studies report minimal change or no improvement in LDL particle size [17,18,21,36].

Mechanistic studies also suggest effects beyond standard lipid panels, including possible regulation of HDL metabolism via angiopoietin-like protein-3-related pathways, metabolic improvements in MASLD populations treated with dapagliflozin, and tissue-level changes in lipid handling, such as altered adipose lipid transport [16,24,30].

Risk of Bias and Overall Quality

A summary of risk-of-bias categories across the included primary records is presented in Table 6.

**Table 6:** Overall risk of bias summary across the included records (N = 32) Overall risk of bias across the included primary studies (N = 32). RoB 2 = low risk (N = 12), RoB 2 = some concerns (N = 11), ROBINS-I = moderate risk of bias (N = 7), ROBINS-I = serious risk of bias (N = 2). Protocol and design records and secondary evidence were not eligible for outcome-level risk of bias assessment and were retained for transparency only [8,47-49].

Tool/Study type	Overall category	Number of records
RoB 2 (RCTs)	Low risk	12
RoB 2 (RCTs)	Some concerns	11
ROBINS-I (Observational)	Moderate	7
ROBINS-I (Observational)	Serious	2
Not assessed	Not assessed (protocols and evidence syntheses)	4

Risk of bias in randomized and interventional studies is summarized in Table 7.

**Table 7:** Risk of bias in randomized and interventional studies (RoB 2) (N = 23) RoB 2 domains: D1, randomization process; D2, deviations from intended interventions; D3, missing outcome data; D4, measurement of the outcome; D5, selection of the reported result. Ratings: Low risk/Some concerns/High risk

Study (Reference)	Study Design	Tool Used	D1	D2	D3	D4	D5	Overall Risk
Kahl et al. (2020) [15]	Randomized, double-blind, placebo-controlled phase 4 trial	RoB 2	Some concerns (randomization details insufficiently reported in extractable text)	Some concerns (insufficient reporting to judge deviations from intended interventions)	Some concerns (attrition/missing outcome handling not accessible)	Low	Some concerns	Some concerns
Lauritsen et al. (2022) [16]	Randomized, double-blinded, placebo-controlled crossover (empagliflozin vs placebo) with washout	RoB 2	Some concerns (sequence generation/allocation concealment not fully extractable)	Low (double-blind placebo cross-over described)	Some concerns	Low	Some concerns	Some concerns
Fadini et al. (2017) [17]	Randomized placebo-controlled trial; single-blind; computer-generated sequence; endpoint evaluation by blinded assessors	RoB 2	Some concerns	Some concerns (single-blind → potential performance bias, although outcomes are lab-based)	Some concerns (33 randomized/31 completed stated, but limited accessible detail on missingness handling)	Low (endpoint evaluation by blinded people; objective measures)	Low (trial registration reported)	Some concerns
Hayashi (2017) [18]	Prospective randomized open-label trial (dapagliflozin vs sitagliptin add-on); trial registered (UMIN)	RoB 2	Some concerns (randomization method/concealment not clearly extractable from accessible text)	Some concerns (open-label → possible deviations; although lipid endpoints are objective)	Some concerns (attrition/missing data handling not clearly stated in extractable text)	Low (objective lipid outcomes; measurement bias less likely)	Low (trial registration reported)	Some concerns
Zinman et al. (2015) [19]	Randomized, double-blind, placebo-controlled cardiovascular outcomes trial	RoB 2	Low (computer-generated random sequence; interactive voice/web response system; stratified randomization described in article text)	Low (double-blind placebo-controlled)	Low (high completion and vital status availability reported)	Low (events/deaths prospectively adjudicated by clinical-events committees)	Low (ClinicalTrials.gov registration NCT01131676 stated)	Low
Bauer et al. (2025) [20]	Randomized, double-blind, placebo-controlled, parallel-group phase IV trial (lipidomics analysis of EmDia samples; NCT02932436)	RoB 2	Not yet extracted	Not yet extracted	Not yet extracted	Not yet extracted	Not yet extracted	Not yet extracted
Emanuelsson et al. (2025) [21]	Post-hoc analyses of 2 randomized, double-blinded, placebo-controlled trials (Empire HF: 190 HF patients; SIMPLE: 90 T2D patients), 1:1 to empagliflozin vs placebo for 12 weeks	RoB 2 (randomized trials)	Some concerns (randomization process details not described beyond 1:1 randomization)	Low (double-blinded, placebo-controlled)	Low (reports trials completed as planned)	Low (objective lab outcomes measured using standard methods)	Some concerns (explicitly described as exploratory/post-hoc)	Some concerns
Bays et al. (2017) [22]	Post hoc analysis of 10 phase 3, placebo-controlled studies (dapagliflozin 10 mg vs placebo; 24 weeks)	RoB 2 (not fully applicable to pooled post-hoc analysis)	Some concerns (not assessable from the pooled report)	Some concerns	Some concerns	Low (lab lipids)	High (post-hoc/selection risk)	High
Dixon et al. (2021) [23]	Post-hoc analysis of CANA-HF; CANA-HF described as a prospective randomized controlled study (canagliflozin vs sitagliptin); 35/36 had baseline + 12-week lipids	RoB 2	Some concerns (randomization details not in abstract)	Some concerns (blinding/deviations not stated in the abstract)	Low (35/36 complete lipid data)	Low (objective serum lipids)	Some concerns (explicit post-hoc analysis)	Some concerns
Hiruma et al. (2021) [25]	Prospective randomized; open-label, blinded-endpoint; randomization via computer-based dynamic allocation	RoB 2	Low	Some concerns (open-label)	Some concerns (exclusions/analysis set details suggest possible missingness impact)	Low (blinded endpoint stated)	Some concerns (protocol/analysis plan not fully checked here)	Some concerns
Burggraaf et al. (2022) [26]	Placebo-controlled randomized proof-of-concept; randomly and double-blindly allocated to dapagliflozin vs placebo for 12 weeks (n=14 men)	RoB 2	Some concerns (sequence/concealment not detailed in abstract)	Low (double blind)	Some concerns (missing data handling not described in the abstract)	Low (objective lab outcomes)	Some concerns (registration/analysis plan not assessed here)	Some concerns
Wazir et al. (2022) [27]	Randomized, open-label clinical trial; 59 adults randomized 1:1 to empagliflozin 10 mg vs 25 mg add-on; fasting lipid profile measured at baseline and 12 weeks	RoB 2	Some concerns (method of random sequence/concealment not described in accessible text)	Some concerns (open-label stated)	Some concerns (attrition/missing handling not clearly described in accessible text)	Low (objective lab measurement)	Some concerns (no protocol/registry details seen in accessible text)	Some concerns
Jojima et al. (2021) [28]	Randomized, active-controlled, open-label trial; 51 patients allocated 2:1 to empagliflozin 10 mg/day vs standard therapy for 12 weeks	RoB 2	Some concerns (randomization procedure details not provided in the abstract)	Some concerns (open-label stated)	Some concerns (missing-data handling not stated in the abstract)	Low (objective sterols + lipid labs)	Some concerns (analysis plan/registration not stated in abstract)	Some concerns
Najim et al. (2025) [29]	Interventional open-label randomized clinical trial; 41 T2DN patients “divided…randomly” into DAPA 5 mg/day vs EMPA 10 mg/day for 16 weeks; lipids measured at baseline and week 16	RoB 2	Some concerns (randomization method/concealment not detailed)	Some concerns (open-label stated)	Some concerns (missing-data handling not clearly stated in the accessible section)	Low (objective lab outcomes; analyzer methods described)	Some concerns (no preregistered analysis plan seen in accessible section)	Some concerns
Fukada et al. (2025) [30]	Randomized controlled trial of dapagliflozin in MASLD with T2D (as stated in the article PDF)	RoB 2	Some concerns (randomization process details not clearly extractable from accessible text segments)	Some concerns (blinding/deviation-control not clearly described in accessible text segments)	Some concerns (missing-data handling not clearly described in accessible text segments)	Low (outcomes largely objective labs/imaging-based in RCT context, per trial report)	Some concerns (analysis plan/selection reporting not clearly verifiable from accessible text segments)	Some concerns
Langslet et al. (2020) [31]	Post-hoc analysis of EMPA-REG OUTCOME: participants randomized to empagliflozin 10/25 or placebo; LDL-C subgroup cutoffs “established post-hoc”; modified ITT used; outcomes adjudicated by Clinical Events Committees	RoB 2	Some concerns (subgroup comparisons not stratified by LDL-C; baseline subgroup imbalances possible)	Some concerns (deviations control/blinding not stated in accessible text)	Some concerns (baseline LDL-C missing for some participants; subgroup data availability issue)	Low (CV outcomes prospectively adjudicated)	High (explicit post-hoc subgroup cutoffs and post-hoc analysis)	High
Khanna (2025) [42]	Prospective parallel-group open-label study; randomized (computer-generated block randomization) dapagliflozin vs vildagliptin; per-protocol analysis; 383 randomized, 248 completed	RoB 2	Some concerns	High	High	Low	Some concerns	High.
Moura et al. (2021) [32]	Sub-analysis of a randomized, double-blind, placebo-controlled trial	RoB 2	Low	Low	Some concerns	Low	Some concerns	Some concerns
Li et al. (2025) [33]	Prospective, single-center, randomized, open-label parallel trial	RoB 2	Low	Some concerns	Some concerns	Low	Some concerns	Some concerns
Kabir et al. (2023) [34]	Placebo-controlled intervention study; group assignment by convenience sampling (not truly randomized)	RoB 2	High	Some concerns	Some concerns	Low	Some concerns	High
Nagao et al. (2024) [35]	Multicenter, randomized, open-label, active-controlled trial	RoB 2	Low	Some concerns	Some concerns	Low	Some concerns	Some concerns
Rau et al. (2021) [36]	Randomized, double-blind, placebo-controlled study (lipoprotein subfractions)	RoB 2	Low	Low	Some concerns	Low	Some concerns	Some concerns
Ejiri et al. (2022) [37]	Randomized controlled trial (UMIN-CTR registered); prespecified sub-analysis	RoB 2	Low	Some concerns	Some concerns	Low	Low	Some concerns

Risk of bias in observational and nonrandomized studies is summarized in Table 8.

**Table 8:** Risk of bias in observational and nonrandomized studies (ROBINS-I) (N = 9) ROBINS-I judgments are summarized using a harmonized D1–D5 structure: D1, confounding/nonrandomized allocation; D2, selection/deviations from intended interventions; D3, missing outcome data; D4, outcome measurement; D5, selective reporting. Overall ROBINS-I categories: Low/Moderate/Serious/Critical

Study (Reference)	Study Design	Tool Used	D1	D2	D3	D4	D5	Overall Risk
Kamijo et al. (2019) [38]	Multicenter prospective single-arm pre–post study (canagliflozin 100 mg for 12 weeks; n=22).	ROBINS-I	Serious	No info	No info	No info	No info	Serious (non-comparative pre–post → high confounding risk)
Doğanay et al. (2023) [39]	Single-center retrospective study; SGLT2i vs propensity-matched control; fasting samples compared at baseline and week 12; excluded “missing clinical data”.	ROBINS-I	Moderate	Moderate	Moderate	Low	No info	Moderate
Ku et al. (2021) [40]	3-year open-label prospective observational study; empagliflozin vs dapagliflozin add-on (n=362); trial number listed (NCT03748810).	ROBINS-I	Serious	Moderate	No info	No info	Moderate	Serious
Grubić Rotkvić et al. (2023) [41]	Prospective observational study; patients divided into planned SGLT2i vs DPP-4 inhibitor groups; baseline and 6-month follow-up (n=64).	ROBINS-I	Serious	Moderate	No info	No info	No info	Serious
Lim et al. (2022) [43]	Retrospective study comparing dapagliflozin vs empagliflozin using propensity score matching.	ROBINS-I	Moderate	Moderate	No info	No info	No info	Moderate
Pahlavan et al. (2025) [44]	12-week prospective single-center clinical trial; empagliflozin added to metformin (80 enrolled; 74 completed); trial registration reported (IRCT trial/73164).	ROBINS-I	Serious	Moderate	Moderate	Low	Low	Serious
Cokro et al. (2025) [45]	Real-world comparative study described as a retrospective cohort; reports handling missing data with imputation in methods.	ROBINS-I	Moderate	Moderate	Moderate	No info	No info	Moderate
Gogoleva et al. (2023) [46]	“Open cohort controlled prospective study”; 60 patients; dapagliflozin 10 mg added to metformin; 6-month follow-up with lipid panel in dynamics.	ROBINS-I	Serious	Moderate	No info	Low	No info	Serious
Liu et al. (2024) [24]	Single-center, open-label, nonrandomized, prospective (metformin vs canagliflozin; 12 weeks); baseline differences reported.	ROBINS-I	Serious/Critical (confounding likely: nonrandomized; baseline differences)	Serious	Low (interventions clearly defined)	Serious (open-label; treatment decisions not randomized)	Some concerns (missing data handling not clear in accessible excerpt)	Serious

Methodological quality of the included systematic reviews/meta-analyses was appraised using AMSTAR 2 and is summarized in the Appendix. Rationale/design protocol papers were listed for transparency and are presented in the Appendix. Outcome-level risk of bias not applicable.

Discussion

Liver-Adipose Pathways 

Kahl et al. [15] show that empagliflozin reduces liver fat in a randomized, placebo-controlled trial. This matters because liver fat is closely linked to atherogenic dyslipidemia, and improving liver fat and metabolism can affect how the body makes and clears lipoproteins. Lauritsen et al. [16] also add that SGLT2 inhibitors can change how lipids move in fat tissue, suggesting that shifts in tissue lipid handling can influence blood lipids, but not always in a predictable way for LDL-C or triglycerides in every group.

“HDL Quantity” Versus “HDL Quality” and Subfraction Remodeling

One reason for mixed results in the literature is that some studies look at lipid concentrations (HDL-C, LDL-C, TG), while others focus on function or subfractions. Fadini et al. [17] address this by studying HDL particle features and cholesterol efflux with dapagliflozin, showing that changes in HDL-C do not always mean better HDL function. Hayashi et al. [18] find that dapagliflozin reduces small dense LDL-C and raises HDL2-C compared to sitagliptin, suggesting SGLT2 inhibitors may lead to a less atherogenic lipoprotein profile, even if standard lipid results seem minor. Overall, these studies suggest that benefits are clearer when looking at particle composition rather than just traditional lipid levels, but the results depend on which aspect of lipids is measured.

Outcome-trial context - benefits can be dissociated from lipid panel direction: Zinman et al. [19] gave strong clinical evidence that empagliflozin improves heart outcomes and survival, even though changes in lipid levels are small. This shows that changes in lipid panels are secondary markers, not the main reason for the benefits. Thus, small increases in LDL-C in some studies should not be seen as harmful if the overall outcomes are positive.

Deep phenotyping: lipidomics and “mixed-direction” remodeling: Bauer et al. [20] went beyond standard lipid panels and showed that empagliflozin changes the overall lipid profile. Lipidomics can detect changes in many types of lipids at once, some good, some possibly not, helping explain why regular lipid panels can look neutral or even conflicting. This suggests SGLT2 inhibitors affect lipids in complex ways, not always in a single positive direction.

Heart failure and high-risk contexts, neutral averages despite metabolic shifts: Emanuelsson et al. [21] find that in people with type 2 diabetes and heart failure, changes in blood lipids are small after adjusting for other factors, even though metabolism improves. This is different from studies showing subfraction improvements and highlights how patient factors, treatments, and other conditions can affect lipid results.

Phenotype stratification and comparator dependence in conventional lipid endpoints: Bays et al. [22] show that dapagliflozin's effects on lipids are small and depend on the patient's starting triglyceride and HDL levels. In a complex heart failure group, Dixon et al. [23] found no major differences in standard lipid measures between canagliflozin and sitagliptin, showing that the choice of comparison drug and other health issues can hide expected changes. Liu et al. [24] connect canagliflozin's lipid effects to ANGPTL3, suggesting a possible reason why triglyceride-rich lipoproteins change in some groups but not others. Hiruma et al. [25] focus on heart fat and metabolism, supporting the idea that metabolic changes can happen even if lipid panels show only small shifts.

Postprandial biology - what fasting lipids can miss: Burggraaf et al. [26] showed that even if fasting or post-meal cholesterol and triglycerides do not change much, remnant markers such as apoB48 can improve, suggesting better atherogenic pathways. In contrast, Wazir et al. [27] found little change in standard lipid measures with empagliflozin in practice, which could be due to small study size, short follow-up, or a real lack of effect.

Reconciling LDL variability - absorption markers and agent-specific patterns: Jojima et al. [28] suggest that LDL-C may rise in some settings alongside higher campesterol, which may reflect increased intestinal cholesterol absorption rather than direct proof of causation. This pattern is consistent with later studies in which LDL-C increases were observed as metabolic status improves, indicating that changes in absorption markers can accompany LDL-C changes. In diabetic kidney disease, Najimi et al. [29] reported differing lipid changes with dapagliflozin versus empagliflozin, suggesting that agent-specific effects and disease context may influence results, although attribution remains challenging because of study design and overlapping treatments.

Liver disease phenotypes and trade-offs: Fukada et al. [30] looked at dapagliflozin in metabolic liver disease and found that it works, but also point out possible risks, such as changes in body composition. This means lipid improvements should be considered along with overall metabolic safety. Langslet et al. [31] showed that empagliflozin's heart benefits are steady across different LDL-C levels, so small LDL-C changes do not seem to affect outcomes. Moura et al. [32] also placed lipid results in the wider context of metabolic syndrome, suggesting that lipid changes are just one part of broader body changes over time.

Longer follow-up and co-interventions - attribution becomes harder: Li et al. [33] pointed out that using other treatments such as probiotics with dapagliflozin makes it hard to know if lipid changes are due to SGLT2 inhibitors alone. Kabir et al. [34] saw clearer improvements in standard lipids with dapagliflozin, but these results should be viewed carefully, since big effects in one study can reflect differences in starting treatments, co-interventions, and longer follow-up where therapies may overlap, as well as natural changes over time or other unknown factors.

Atherogenic profiling and the strongest internal context-dependent signal: Nagao et al. [35] demonstrated that atherogenic lipoprotein profiles shift differently depending on the comparator, reinforcing the notion that treatment effects are often relative to the comparator's biology. One of the most directly contrasting findings emerges in Rau et al. [36], where empagliflozin increased LDL-C and apoB without clear improvement in LDL particle size, standing in tension with the subfraction improvements reported earlier by Hayashi et al. [18]. Rather than a contradiction, these findings support a comparator-sensitive and population-specific signal, and they underscore a practical conclusion that qualitative lipoprotein remodeling is not guaranteed. Even when outcome benefits appear preserved, metabolic nuance can remain, and in some contexts, LDL particle burden, including apoB-related burden, may rise as clinical benefits persist.

Heart failure again - neutral lipid signals in controlled comparisons: Ejiri et al. [37] found that, in patients with type 2 diabetes and heart failure, luseogliflozin did not clearly improve certain high-risk lipid profiles compared with voglibose, consistent with the trend that heart failure patients may have weaker or less pronounced lipid changes. Kamijo et al. [38] reported that canagliflozin increases large HDL fractions, suggesting that subfraction changes can occur even when standard lipid results are mixed.

Real-world and longer-term cohorts - direction depends on setting and adjustment: Doğanay et al. [39] saw better lipid results in people with high blood pressure and type 2 diabetes using SGLT2 inhibitors, but because the study looks back at records, other treatment changes could affect the results. Ku et al. [40] compared empagliflozin and dapagliflozin over three years and saw positive lipid trends, but changing medications and how well patients stick to treatment make it hard to be sure of the cause. Grubić Rotkvić et al. [41] added more detail about the heart and metabolism, but since their study is not randomized, it is still hard to draw firm conclusions.

Comparator effects made explicit in head-to-head glucose-lowering trials: Khanna et al. [42] showed that dapagliflozin versus vildagliptin yields different lipid trajectories, with findings that can make the SGLT2 arm appear better (e.g., HDL-C) or worse (e.g., LDL-C) depending on which endpoint is prioritized, highlighting comparator-driven interpretation. Lim et al. [43] reinforced that, in longer-term real-world follow-up, LDL-C can trend higher, particularly with empagliflozin, again consistent with earlier absorption-marker hypotheses, and emphasize that LDL direction may change with time horizon and setting.

NAFLD/MAFLD cohorts and real-world paired analyses: Pahlavan et al. [44] reported improvements in metabolic profile and liver function tests in MAFLD populations treated with empagliflozin, supporting the hypothesis that a hepatic phenotype can favor more consistent beneficial lipid effects. Cokro et al. [45] provided paired real-world comparative data showing broad cardiometabolic improvements with both dapagliflozin and empagliflozin while illustrating that apparent between-drug differences may attenuate after adjustment, an important methodological reminder. Gogoleva et al. [46] similarly reported favorable lipid and metabolic shifts in obese patients with type 2 diabetes, consistent with a model in which changes in body composition and adipose biology contribute meaningfully to lipid outcomes.

Chen et al. [47] helped explain conflicting study results by showing that, overall, there are usually small drops in triglycerides and rises in HDL, while LDL changes are small and can differ between studies. Shigiyama et al. [48,49] pointed out that some papers included are just study designs, not results, so it is important not to count them as outcome data when looking at lipid effects.

Summary

Overall, the studies show that SGLT2 inhibitors most often lower triglycerides a bit and raise HDL, sometimes with changes in lipoprotein subfractions or remnant markers. However, LDL-C results are mixed and depend on patient type, comparison drug, follow-up time, other lipid treatments, and how lipids are measured. This means SGLT2 inhibitors should mainly be seen as treatments for the heart, the kidney, and metabolism, with lipid changes as secondary effects to watch, especially if apoB or LDL-C goes up, rather than as the main reason for their benefit.

## Conclusions

SGLT2 inhibitors have a largely neutral to mildly favorable lipid profile in type 2 diabetes mellitus. LDL-C and total cholesterol are generally unchanged, HDL-C increases modestly, and triglycerides tend to decrease, particularly in atherogenic dyslipidaemia. Overall, these findings support SGLT2 inhibitors as cardiometabolic therapies without a consistent adverse lipid penalty.

Future studies should prioritize longer follow-up randomized trials and standardized advanced lipid reporting, with apoB as the most clinically actionable marker of atherogenic particle burden, alongside remnant cholesterol and HDL functional measures. Head-to-head and real-world studies are also needed to clarify agent-specific effects and optimize combination strategies.

## Appendices

Appendix Table S1 and Appendix Table S2 provide the risk-of-bias-related summaries for evidence syntheses and protocol papers.

**Table 9:** S1: Methodological quality of systematic reviews and meta-analyses (AMSTAR 2) AMSTAR 2-mapped domains: D1, protocol registration; D2, search comprehensiveness; D3, duplicate screening/extraction; D4, risk-of-bias assessment and use; D5, publication bias and conflicts of interest. Overall confidence reflects AMSTAR 2 guidance.

Study (Reference)	Study Design	Tool Used	D1	D2	D3	D4	D5	Overall Risk/Confidence
Storgaard et al. (2016) [8]	Systematic review + meta-analysis of double-blind RCTs	AMSTAR 2	Yes (PROSPERO CRD42014008960)	Yes (Cochrane Library/MEDLINE/EMBASE/Science Citation Index/WHO Trial Search DB + search string + last update Oct 2015)	Yes (trial selection by 2 reviewers; extraction by 3 reviewers independently)	Yes (Cochrane RoB tool; meta-analysis methods described)	Yes (Egger/Harbord + funnel plots; Funding statement “no specific funding”; COI declared)	Moderate–High confidence
Chen et al. (2021) [47]	Systematic review + network meta-analysis (24-week)	AMSTAR 2	Yes (PROSPERO CRD42020198516)	Partial	Yes (Two researchers independently screened/extracted)	Yes (Cochrane Handbook RoB tool)	Yes	Low–Moderate confidence)

**Table 10:** S2: Protocol/rationale papers (listed for transparency; outcome-level RoB not applicable) Protocol/rationale papers are listed for transparency and describe planned methods but do not report outcome results; therefore, outcome-level risk-of-bias judgments are not applicable (N/A).

Study (Reference)	Study Design	Tool Used	D1	D2	D3	D4	D5	Overall Risk
Shigiyama et al. (2019) [48]	Rationale & design / Protocol paper (no results/outcomes reported)	Not applicable (no RoB for outcomes)	N/A (protocol/design)	N/A (protocol/design)	N/A (protocol/design)	N/A (protocol/design)	N/A (protocol/design)	N/A — Protocol/Design paper (no outcomes)
Shigiyama et al. (2018) [49]	Rationale & design / Protocol paper (no results/outcomes reported)	Not applicable (no RoB for outcomes)	N/A (protocol/design)	N/A (protocol/design)	N/A (protocol/design)	N/A (protocol/design)	N/A (protocol/design)	N/A — Protocol/Design paper (no outcomes)
